# From perception to response: translational progress implementation challenges of wearable non-invasive continuous vital sign monitoring in early warning for inpatients

**DOI:** 10.3389/fphys.2026.1925052

**Published:** 2026-09-14

**Authors:** Chunli Dong, Junxiao Yang

**Affiliations:** Shandong First Medical University, Jinan, Shandong, China

**Keywords:** clinical deterioration, continuous vital-sign monitoring, early warning, hospitalized patients, wearable monitoring

## Abstract

**Background:**

Intermittent ward observations may miss short-lived physiological deterioration, and failures in observation, documentation, scoring, or escalation can widen this surveillance gap. Wearable, non-invasive continuous vital-sign monitoring may narrow the gap, but its translational value depends on whether continuous data are converted into timely and sustainable clinical response.

**Objective:**

To map evidence on wearable continuous or near-continuous vital-sign monitoring for inpatient deterioration detection, alerting, clinical response, workflow integration, and implementation.

**Methods:**

We conducted a PRISMA-ScR-informed scoping review. PubMed, Embase, Web of Science, and Scopus were searched from January 2010 to May 2026. Eligible reports evaluated wearable, non-invasive monitoring in non-ICU inpatient settings and reported technical, clinical, workflow, user-experience, or implementation outcomes. Findings were synthesized along a monitor–response pathway.

**Results:**

Of 6,093 database records identified, 32 studies were included. Evidence was concentrated in signal acquisition, technical feasibility, abnormality detection, and user experience. Continuous monitoring identified oxygen desaturation and abnormal respiratory-rate episodes not captured by intermittent observations, but respiratory-rate performance varied with the sensor and comparator method. Reporting of standard care, alarm configuration, bedside reassessment, escalation procedures, and response fidelity was incomplete. Observational and before-after studies reported favorable clinical outcomes in selected settings, whereas randomized and pragmatic trials were heterogeneous and several pilot or feasibility studies were not designed or powered for uncommon clinical endpoints. One preliminary prediction-modeling study was identified.

**Conclusion:**

The evidence base is more mature for the afferent limb of monitoring and detection than for the efferent limb of clinical assessment, intervention, escalation, and outcome improvement. Wearable monitoring should be evaluated as a complex monitor-response intervention, with explicit standard care, alarm logic, response protocols, implementation support, and response fidelity.

## Highlights

- This scoping review maps 32 studies across a monitor–response pathway.
- Wearable monitoring detects intermittent abnormalities missed by routine ward observations.
- Evidence is strongest for feasibility, abnormality detection, and patient acceptability.
- Hard clinical outcomes remain inconsistent across randomized and real-world studies.
- Alert burden, connectivity, and workflow integration are key barriers to clinical translation.

## 1 Introduction

Clinical deterioration in hospitalized patients is often preceded by changes in vital signs, but the prognostic contribution of individual parameters is not equivalent. Respiratory rate is particularly important in early deterioration assessment, while oxygen saturation and heart rate can identify specific hypoxemic or circulatory abnormalities (Schein et al., 1990; Kause et al., 2004; Duus et al., 2018; Haahr-Raunkjaer et al., 2022). Ward surveillance therefore combines vital-sign observations with early warning systems such as the Modified Early Warning Score (MEWS), the National Early Warning Score (NEWS), and its updated version, NEWS2, which standardize reassessment and escalation while remaining dependent on clinical judgement (Subbe et al., 2001; Smith et al., 2013; Royal College of Physicians, 2017).

General wards increasingly care for older, multimorbid, and postoperative patients outside higher-dependency units. Scheduled spot checks can leave several hours without physiological observations, particularly overnight or soon after surgery, but the observation gap is not caused by measurement intervals alone. Incomplete observation, documentation, score calculation, and adherence to escalation protocols can also delay recognition and response (Duus et al., 2018; Weenk et al., 2019; Haahr-Raunkjaer et al., 2022).

Respiratory rate illustrates both the value and the measurement difficulty of ward surveillance. It is a clinically important marker of deterioration, yet routine manual counts are affected by observation duration, rounding, patient awareness, and inter-observer variability. Wearable estimates are also vulnerable to motion, posture, sensing algorithms, and data loss. Disagreement between a wearable device and standard care may therefore reflect the sensor, the comparator, or both, rather than comparison with an error-free reference standard (Downey et al., 2019; Breteler et al., 2020).

Wearable, non-invasive continuous monitoring has been proposed as a way to narrow the observation gap without converting general wards into intensive-care-like environments. Patch-based sensors, wearable multiparameter devices, and mobile telemetry systems can acquire heart rate, respiratory rate, oxygen saturation, temperature, blood pressure, or multimodal data while preserving mobility. Early studies nevertheless reported artefact, skin irritation, battery limitations, connectivity loss, and parameter-specific missing data (Weenk et al., 2017; Downey et al., 2018b; Leenen et al., 2021).

As research has matured, the central question has shifted from whether signals can be collected to what happens after a trend or alert is generated. Systems have used fixed thresholds, persistence rules, individualized limits, mobile notifications, central displays, alarm-free trend review, and algorithmic scores. These configurations alter sensitivity, positive predictive value, workload, and actionability. Their effect also depends on who reviews the data, whether intermittent observations continue, how bedside reassessment is performed, and whether the alert is connected to an existing MEWS, NEWS2, rapid-response, or medical escalation pathway (Leenen et al., 2022b; Peelen et al., 2023; Mølgaard et al., 2025).

Clinical evidence has become more outcome-oriented, but interpretation is constrained by design and statistical capacity. Observational and quality-improvement reports have described fewer complications, rapid-response calls, unplanned intensive care transfers, shorter stays, or lower costs after implementation (Verrillo et al., 2019; Eddahchouri et al., 2022; Vroman et al., 2023; Rowland et al., 2024). Randomized and pragmatic trials have reported more selective effects on physiological deviation or patient-centered outcomes, and several studies were designed for feasibility rather than rare outcomes such as death or cardiac arrest (Posthuma et al., 2024; Mølgaard et al., 2025; Khanna et al., 2026). Prediction modeling was represented by only one included report and therefore remains a preliminary extension of the mapped evidence (Scheid et al., 2025).

Implementation is consequently part of the intervention rather than a downstream operational detail. Patients often value perceived safety but do not want technology to replace direct nursing contact. Clinicians value earlier trend visibility but report concerns about false alerts, device maintenance, workload, electronic health record integration, training, and accountability for response (van Rossum et al., 2021; Weenk et al., 2020; Leenen et al., 2022a; Becking-Verhaar et al., 2023; van Noort et al., 2024).

Because devices, monitored parameters, alarm strategies, clinical response pathways, and outcome definitions vary substantially, the translational maturity of wearable inpatient monitoring remains difficult to judge. This scoping review maps the evidence across a harmonized monitor-response pathway: clinical data acquisition; signal processing and data management; modeling and interpretation; alerting and notification; clinical response and action; and implementation and sustainability. It specifically examines the imbalance between the afferent limb of sensing and detection and the efferent limb of assessment, intervention, escalation, and outcome improvement.

## 2 Methods

### 2.1 Study design

**Figure 1:**
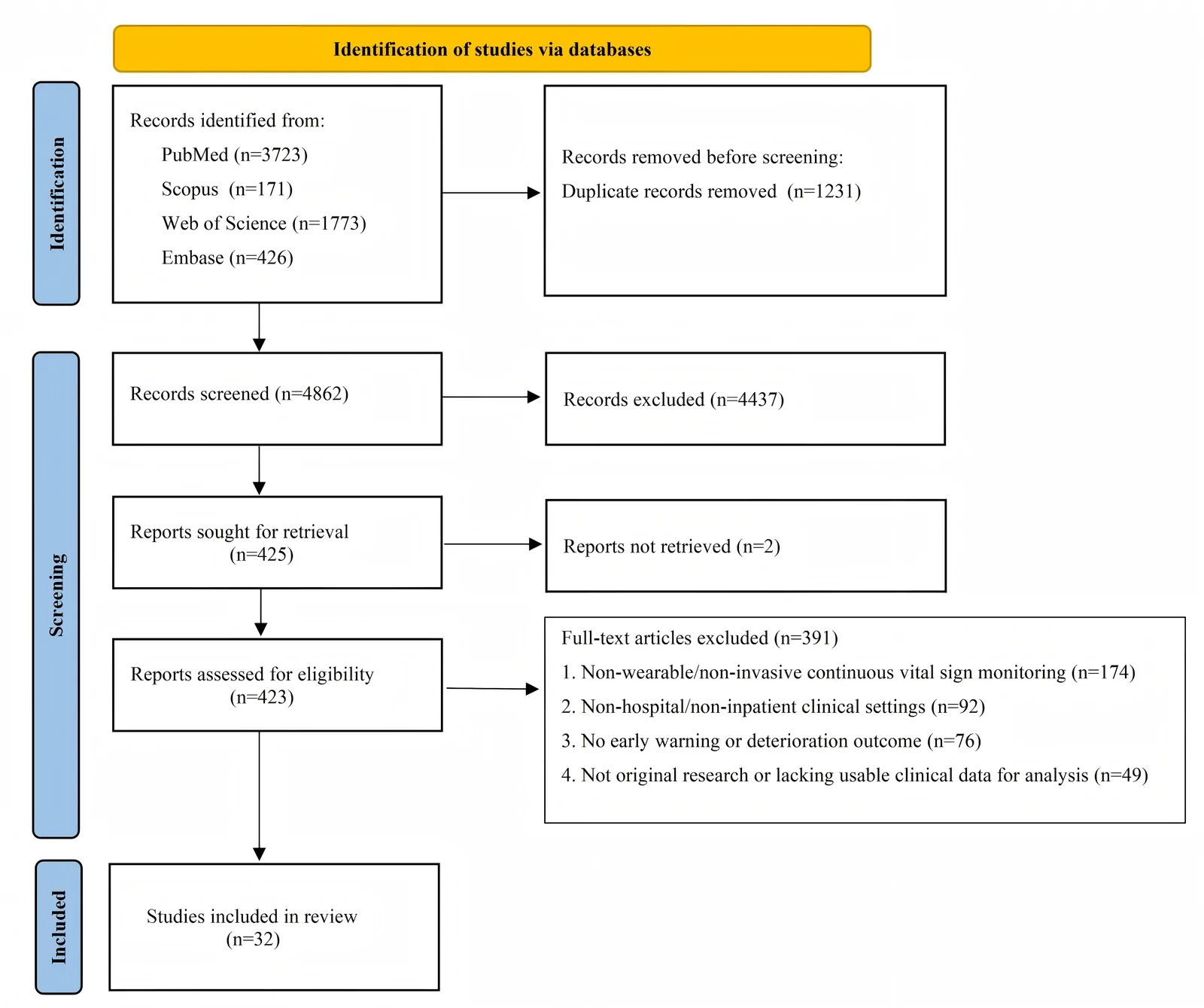
PRISMA flow diagram of study selection.

This scoping review maps the translational evidence for wearable non-invasive continuous vital sign monitoring to detect deterioration in hospitalized patients. We chose a scoping approach because the existing literature is heterogeneous in device types, signals monitored, and clinical pathways. This review follows the methodological framework initially proposed by Arksey and O’Malley in 2005 and further refined by Levac et al. in 2010, while also adhering to the latest guidelines for scoping reviews and evidence mapping (Campbell et al., 2023; Munn et al., 2018; Peters et al., 2024). This study’s report followed the extended PRISMA-ScR statement for scoping reviews and used a PRISMA 2020 flow diagram to document the study screening process (Page et al., 2021; Tricco et al., 2018). The detailed flow diagram is shown in **Figure 1**.

### 2.2 Questions and eligibility criteria

The eligibility criteria and data-charting framework were specified before screening; however, no formal protocol was registered or publicly archived. The review question and eligibility criteria followed the population-concept-context framework recommended for scoping reviews (Peters et al., 2024). The population comprised hospitalized inpatients. The concept was wearable, non-invasive continuous or near-continuous monitoring of heart rate, respiratory rate, oxygen saturation, temperature, blood pressure, or multimodal physiological parameters. Contexts included general medical, surgical, postoperative, step-down, and emergency observation areas outside the ICU. Continuous monitoring was operationally defined as automatic wearable acquisition across a monitoring episode without reliance on nurse-performed spot checks. Near-continuous monitoring was defined as frequent wearable acquisition intended to support trend review, alerting, or escalation, allowing interruptions caused by removal, battery status, connectivity, artefact, or transmission intervals. Sampling frequency, averaging windows, and alert logic were charted as reported by the original studies.

### 2.3 Search strategy

From January 2010 to May 2026, a systematic literature search was conducted in four electronic databases: PubMed, Embase, Web of Science Core Collection, and Scopus. These four databases were selected to provide broad biomedical, clinical, and multidisciplinary coverage. PubMed and Embase were used to capture biomedical and clinical studies, whereas Web of Science Core Collection and Scopus were included to broaden multidisciplinary coverage relevant to digital health, monitoring technologies, and implementation research. The search strategy was designed to be sensitive rather than narrowly specific by combining controlled vocabulary and free-text terms for wearable devices, vital signs, inpatient settings, clinical deterioration, alerting, workflow, feasibility, usability, and implementation. No language or publication-type restriction was applied at the search stage. We also screened the reference lists of included studies and relevant reviews to identify additional eligible records. The retrieved evidence set included early formative inpatient wearable-monitoring studies that met the review criteria, including pilot, validation, feasibility, and early clinical implementation reports. However, no systematic grey-literature search, trial-registry search, conference-proceeding search, CINAHL search, IEEE Xplore search, or forward citation tracking was conducted. Therefore, the review should be interpreted as a map of peer-reviewed clinical literature indexed in the selected databases, supplemented by backward reference checking.

The lower date limit of January 2010 was chosen *a priori* to capture contemporary wearable and wireless inpatient monitoring technologies while excluding older bedside telemetry, wired monitoring, and intensive-care-oriented monitoring systems that were outside the concept of this review. Although the search covered records from 2010 onward, the final included studies were published from 2017 to 2026. This gap reflects the application of the eligibility criteria rather than an additional *post hoc* date restriction: earlier records retrieved by the search did not meet the combined criteria of wearable, non-invasive, continuous or near-continuous vital-sign monitoring in non-ICU inpatient settings with extractable clinical, technical, workflow, or implementation outcomes.

The search strategy was developed with reference to PRISMA-S and the PRESS guideline to support transparent and reproducible reporting (McGowan et al., 2016; Rethlefsen et al., 2021). Reference lists of included studies and relevant reviews were screened manually. No additional records were identified through backward reference checking; therefore, no additional studies were included through this process. For PubMed, four predefined concepts were linked using AND; synonyms within each concept were combined using OR, using Medical Subject Headings and title/abstract terms.

(“Wearable Electronic Devices”[Mesh] OR “Monitoring, Physiologic”[Mesh] OR wearable*[tiab] OR wireless[tiab] OR biosensor*[tiab] OR sensor*[tiab] OR patch*[tiab] OR “wearable device*”[tiab] OR “wearable sensor*”[tiab] OR “wireless device*”[tiab] OR “wireless sensor*”[tiab] OR “remote monitor*”[tiab] OR “remote monitoring”[tiab] OR “continuous monitor*”[tiab] OR “continuous surveillance”[tiab])

AND

(“Vital Signs”[Mesh] OR “vital sign”[tiab] OR “vital signs”[tiab] OR “heart rate”[tiab] OR “respiratory rate”[tiab] OR “oxygen satu-ration”[tiab] OR SpO_2_[tiab] OR temperature[tiab] OR “blood pres-sure”[tiab] OR “physiological monitoring”[tiab] OR “physiologic monitoring”[tiab])

AND

(“Inpatients”[Mesh] OR “Hospitalization”[Mesh] OR “Hospital Units”[Mesh] OR inpatient*[tiab] OR hospital*[tiab] OR ward*[tiab] OR “general ward*”[tiab] OR “medical ward*”[tiab] OR “surgical ward*”[tiab] OR postoperative[tiab] OR surgery[tiab] OR surgical[tiab])

AND

(deteriorat*[tiab] OR “clinical deterioration”[tiab] OR “early warning”[tiab] OR NEWS[tiab] OR NEWS2[tiab] OR MEWS[tiab] OR “rapid response”[tiab] OR “rapid response system”[tiab] OR “rapid response team”[tiab] OR “ICU transfer”[tiab] OR “unplanned ICU”[tiab] OR “intensive care unit admission”[tiab] OR “patient safety”[tiab] OR complication*[tiab] OR “adverse event*”[tiab] OR “clinical outcome*”[tiab] OR “patient outcome*”[tiab] OR “failure to rescue”[tiab] OR alarm*[tiab] OR alert*[tiab] OR notification*[tiab] OR implementation[tiab] OR workflow[tiab] OR feasibility[tiab] OR acceptability[tiab] OR usability[tiab] OR experience*[tiab] OR perception*[tiab] OR perspective*[tiab] OR nurs*[tiab])

A publication date filter was applied using PubMed’s publication date field: (“2010/01/01”[Date - Publication]: “2026/05/31”[Date - Publication]). No language or publication type restrictions were applied. The same conceptual structure was adapted for Embase, Web of Science, and Scopus, with database-specific syntax modifications (Embase: Emtree terms and.ti,ab,kw fields; Scopus: TITLE-ABS-KEY; Web of Science: TS field). Full search strategies for all databases are provided in **Supplementary Table 1**.

### 2.4 Inclusion and exclusion criteria

Studies were included if they met the following criteria: (1) original clinical studies conducted in hospitalized patients; (2) evaluations of wearable, non-invasive devices used for continuous or near-continuous monitoring of one or more vital signs-including heart rate, respiratory rate, oxygen saturation, temperature, blood pressure- or multimodal physiological parameters; (3) conducted in inpatient settings such as general wards, postoperative units, step-down units, or emergency observation areas; (4) reporting clinical, technical, workflow, or implementation outcomes related to early deterioration detection or escalation of care.

Studies meeting any of the following criteria were excluded: (1) those focusing on invasive monitoring, relying solely on intermittent manual vital sign observations, using non-wearable monitoring systems, consumer-grade fitness trackers not clinically deployed, non-inpatient or non-hospital settings, or limited to laboratory validation; (2) editorials, reviews, research protocols, conference abstracts lacking sufficient data, or duplicate publications; (3) Studies without extractable clinical, technical, workflow, or implementation data.

### 2.5 Study screening methods

All literature records were imported into the reference management software EndNote. Prior to screening, duplicate records were removed using a structured deduplication method in accordance with the established multi-database workflow (Bramer et al., 2016). Two independent researchers completed the screening of literature titles and abstracts, followed by full-text assessment of literature that potentially met the inclusion criteria. Discrepancies were resolved through discussion, and literature on which no consensus was reached was adjudicated by a third senior researcher. Reasons for full-text exclusion were documented and summarized in the PRISMA flow diagram.

### 2.6 Data extraction and charting

Data were charted with a standardized form that was pilot-tested on a subset of reports and refined during extraction (Pollock et al., 2023). In addition to study design, setting, population, device, parameters, technical performance, outcomes, and limitations, we extracted the intervention surrounding the technology: pre-existing standard care; continuation of intermittent observations; who received, reviewed, or responded to data; integration with MEWS, NEWS2, rapid-response, or local escalation systems; bedside reassessment and escalation requirements; new protocols, training, implementation support, and co-interventions; and adherence or response fidelity. Alarm data included physiological versus technical alerts, parameter thresholds, persistence requirements, annunciation delays, patient- or ward-specific adjustment, delivery route and location, alerts per patient-day or monitoring-day, positive predictive value or true-positive rate, the adjudication definition, and resulting assessments or interventions. For comparative studies, we charted the prespecified primary endpoint, sample-size rationale, and whether the study was designed or powered for the clinical endpoint under discussion. Patient selection, monitoring duration, proportion of admission monitored, reasons for removal or incomplete exposure, and whether alarms were local or remote were also extracted. Unreported items were coded as not reported. Effect estimates, comparator direction, denominators, confidence intervals, and alarm-related numerical data were independently rechecked against the original reports by a second reviewer and discrepancies were resolved by consensus.

### 2.7 Data synthesis

**Figure 2:**
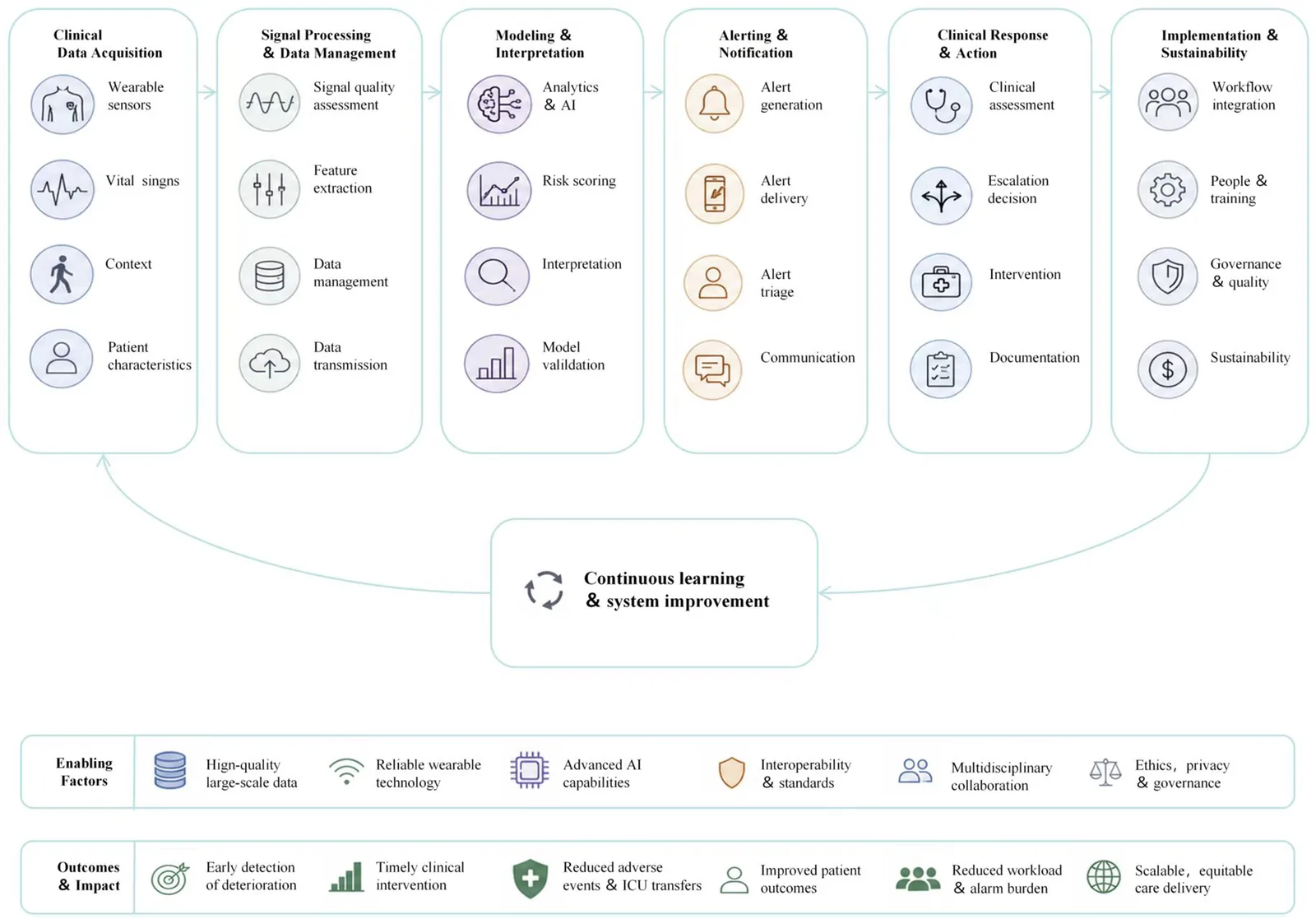
Conceptual monitor-response pathway for wearable continuous vital-sign monitoring in hospitalized inpatients. The first four stages constitute the afferent limb and the final two stages the efferent limb. AI, artificial intelligence; ICU, intensive care unit.

Because devices, comparators, pathways, alarm configurations, and outcomes were heterogeneous, no meta-analysis was conducted. Findings were synthesized narratively across the six stages shown in **Figure 2**: clinical data acquisition; signal processing and data management; modeling and interpretation; alerting and notification; clinical response and action; and implementation and sustainability. The first four stages were considered the afferent limb and the final two the efferent limb of the monitor-response pathway. Implementation findings were interpreted with Proctor’s implementation outcomes, the updated Consolidated Framework for Implementation Research, and the NASSS framework (Damschroder et al., 2022; Greenhalgh et al., 2017; Proctor et al., 2011). No formal critical appraisal or risk-of-bias assessment was undertaken, consistent with the mapping purpose of the review. We did not perform retrospective power calculations; statistical capacity was interpreted from the original study objective, prespecified endpoint, sample-size rationale, achieved sample, and reported uncertainty.

## 3 Results

### 3.1 Study selection

The database search identified 6,093 records before deduplication, including 3,723 from PubMed, 1,773 from Web of Science, 426 from Embase, and 171 from Scopus. After excluding 1,231 duplicate records, a total of 4,862 records underwent title and abstract screening, among which 4,437 records were excluded. Ultimately, 425 reports were retrieved, and full texts could not be obtained for 2 reports. Full-text eligibility assessment was then conducted on the remaining 423 reports. After full-text review, 391 reports were excluded because they did not evaluate wearable or non-invasive continuous vital sign monitoring, were conducted outside hospital or inpatient settings, did not report early warning or deterioration-related outcomes, or were not original studies with extractable clinical data. Finally, 32 studies were included in the evidence synthesis.

### 3.2 Characteristics of included studies

The 32 included studies were published between 2017 and 2026 and covered technical validation, feasibility, comparative effectiveness, implementation, qualitative experience, and predictive modelling. The clinical settings were mainly surgical wards, medical wards, mixed medical–surgical wards, postoperative wards, and surgical step-down units. Most studies evaluated adult postoperative or general ward inpatients, while several studies focused on abdominal surgery, major noncardiac surgery, esophagectomy, or broader medical ward populations.

The included evidence comprised randomized or cluster-randomized studies, stepped-wedge cluster trials, prospective cohorts, before–after studies, propensity-matched observational studies, clinical validation studies, mixed-methods studies, qualitative studies, surveys, and one deep-learning prediction study. Randomized or pragmatic comparative evidence was reported in studies evaluating SensiumVitals, ViSi Mobile, and multimodal postoperative monitoring systems (Downey et al., 2018a, 2020; Khanna et al., 2026; Mølgaard et al., 2025; Posthuma et al., 2024; Weenk et al., 2020). Observational and before–after studies examined clinical outcomes, workflow outcomes, ICU transfers, rapid response team calls, length of stay, and implementation fidelity (Eddahchouri et al., 2022, 2023; Leenen et al., 2021, 2023, 2024; Rowland et al., 2024, 2025; Verrillo et al., 2019; Vroman et al., 2023). Qualitative and survey-based studies described patient, nurse, and physician experiences, implementation barriers, and sustainability (Becking-Verhaar et al., 2023; Downey et al., 2018b, 2022; Kooij et al., 2022; Leenen et al., 2022a, 2022b; Lockhorst et al., 2025; van Noort et al., 2024; van Rossum et al., 2021; Weenk et al., 2017, 2020).

Across studies, the most frequently monitored parameters were heart rate (HR) and respiratory rate (RR). Oxygen saturation (SpO_2_), blood pressure, temperature, and electrocardiographic signals were included less consistently. Devices included ViSi Mobile, HealthPatch, SensiumVitals, Philips Biosensor/Healthdot, Isansys Lifetouch, Nonin WristOx, Masimo Radius-7, EarlySense, VitalConnect, and Biobeat. Chest-worn adhesive patches and wrist-mounted multiparameter monitors were the most common device formats. A detailed summary of study designs, clinical settings, patient populations, devices, and monitored parameters for all included studies is presented in **Supplementary Table 2**.

### 3.3 Technical performance, comparator methods, and data completeness

Technical performance varied by sensing modality, parameter, comparator method, and clinical context. The validation studies did not share a single reference standard. Weenk et al. (2017, 2019) and Downey et al. (2019) compared wearable measurements with routine or study-specific ward observations; respiratory rate in these comparisons was commonly obtained by manual counting, while heart rate, temperature, or oxygen saturation used pulse oximetry, tympanic thermometry, or routine bedside equipment. By contrast, Breteler et al. (2020) compared four systems with an ICU-grade monitor in a surgical step-down unit. Accordingly, the reported agreement represents the performance of each sensor-comparator pair and not comparison with a universally error-free reference.

Heart-rate measurements generally showed more consistent agreement than respiratory-rate measurements within the charted validation studies, but this finding was device- and comparator-specific. Downey et al. (2019) reported a small mean heart-rate bias but wide limits of agreement; respiratory-rate and temperature agreement did not meet the study-specific acceptability limits. The same report noted that manually recorded respiratory-rate distributions were clinically implausible in some observations, illustrating uncertainty in the comparator itself. Breteler et al. (2020) found broadly acceptable heart-rate performance against an ICU-grade reference, whereas respiratory-rate performance differed across devices and HealthPatch overestimated respiratory rate. Weenk et al. (2017, 2019) found that respiratory-rate discrepancies frequently changed calculated MEWS values.

Data completeness also differed by parameter. In Weenk et al. (2017), mean exposure was 61.9 hours for ViSi Mobile and 57.5 hours for HealthPatch, with connectivity failure accounting for 70% of ViSi Mobile artefacts. In the related 60-patient comparison, missing data were 10.1% for ViSi Mobile and 8.4% for HealthPatch (Weenk et al., 2019). Leenen et al. (2021) reported artefact in 19% of heart-rate, 51% of respiratory-rate, and 9% of temperature data, despite a median monitoring duration of 81 hours. Breteler et al. (2020) reported device-specific data loss ranging from approximately 6% to 38%. Thus, ward deployability did not imply uniform parameter accuracy or completeness. Study-level comparator methods and technical limitations are summarized in **Supplementary Table 3**.

### 3.4 Detection, alarm-system design, and alarm burden

Continuous or near-continuous monitoring detected physiological abnormalities not captured by scheduled observations. Duus et al. (2018) reported oxygen saturation below 92% in 98% of postoperative abdominal surgery patients during continuous monitoring compared with 16% during routine EWS observations; episodes below 85% lasting more than 10 minutes occurred in 52%. Weenk et al. (2019) identified 71 ViSi Mobile and 32 HealthPatch high-MEWS periods between routine nurse observations. The studies did not consistently report whether the additionally detected episodes prompted clinical assessment or intervention.

Alarm outcomes varied across system configurations. In Leenen et al. (2021), SensiumVitals used thresholds of 50-120 beats/min for heart rate, 8-24 breaths/min for respiratory rate, and 34.5-38.5 degrees°C for axillary temperature; deviation had to persist for at least 14 minutes. Alerts were delivered to nurse mobile devices and a desktop application, with reminders until acknowledgement. Of 972 alerts, 878 (90.3%) were technical or system alerts and 94 (9.7%) were physiological alerts. Two reviewers retrospectively adjudicated the physiological alerts using the patient condition, nurse MEWS observations, and electronic record: 33 of 94 were true positive (35.1%), 41 were false positive, and 20 were indeterminate. The median burden was 4.5 alerts per patient-day.

Other systems used materially different designs. Posthuma et al. (2024) required deviation for more than 14 minutes before an audible handheld notification and permitted nurses to optimize limits after bedside evaluation. Mølgaard et al. (2025) combined NEWS-derived thresholds with persistence rules and delivered alerts to staff smartphones. Khanna et al. (2026) used alerts for mean arterial pressure below 65 mmHg, oxygen saturation below 90%, and heart rate above 110/min, routed to a central station and nurse phones with escalation to other nurses and the unit manager if unaddressed. In contrast, Leenen et al. (2022b, 2023) used alarm-free trend review: nurses reviewed and documented trends twice per shift, and physicians reviewed them during rounds. Peelen et al. (2023) evaluated an offline algorithmic warning scenario rather than a deployed clinical alert pathway; it generated 0.99 warnings per patient-day, a positive predictive value of 0.007, and a number needed to evaluate of 152. Thresholds, delays, adjustment rules, locations, and resulting actions were incompletely reported in several other studies, which limited cross-study comparison. Study-level alarm thresholds, persistence requirements, alert-delivery routes, alarm burden, predictive values, and resulting clinical actions are summarized in **Supplementary Table 4**.

### 3.5 Standard care and clinical response pathways

Most comparative intervention studies continued intermittent observations alongside continuous monitoring rather than replacing standard care. Downey et al. (2018a, 2020) added SensiumVitals to usual NEWS observations; nurse mobile devices received alerts, and subsequent assessment remained within local nursing and medical protocols. Eddahchouri et al. (2022, 2023) retained the same MEWS and rapid-response protocol before and after ViSi Mobile implementation, including eight-hourly routine observations and more frequent review at higher scores. Continuous data were visible on the patient device and ward displays, but rapid-response activation was not automatic. Mølgaard et al. (2025) added smartphone alerts to standard NEWS care and advised nurses to use the existing escalation pathway; the system did not prescribe a specific intervention. Posthuma et al. (2024) required bedside evaluation after a persistent alert, followed by full MEWS calculation and the hospital escalation protocol when no innocuous cause was found.

The person receiving and acting on data varied. In the Downey studies, the patient nurse received mobile alerts and junior medical staff retained treatment decisions. In Khanna et al. (2026), alerts were sent to the central station and assigned nurse phone and escalated within the nursing hierarchy if not addressed. Leenen et al. (2022b, 2023) deliberately removed automated alerts and required scheduled nurse trend review, documentation, and medical review during rounds. The Weenk et al. (2019) validation study displayed individualized alarm limits but kept clinicians blinded to device results, so detected high-MEWS periods did not form a clinical response intervention. Peelen et al. (2023) compared modeled warning periods retrospectively and likewise did not evaluate real-time clinical response.

Training and implementation support were common co-interventions when reported. Downey et al. (2018a, 2020) provided ward training and adjusted alert settings after excessive initial burden. Leenen et al. (2021) trained 35 nurses, developed key users, and provided bedside support; the later fidelity study used preparation, daily coaching during early implementation, key users, feedback, and quality-improvement cycles (Leenen et al., 2023). Verrillo et al. (2019) combined technology with education, daily coaching, and 24-hour support during early implementation. These components could influence awareness, documentation, escalation, and outcomes independently of the sensor. Response fidelity was measured inconsistently: trend documentation occurred in 80.5% of shifts in Leenen et al. (2022b), overall fidelity was 70.7% and declined over time on the medical ward in Leenen et al. (2023), and 28% of intervention cases in Mølgaard et al. (2025) had no documented nurse acknowledgement or response to an alert. Khanna et al. (2026) could not evaluate planned bedside interventions reliably because documentation was inadequate. **Supplementary Table 5** charts the response pathway and co-interventions.

### 3.6 Statistical capacity and clinical outcomes

Clinical outcomes were reported across pilot, feasibility, randomized, before–after, quality-improvement, and propensity-matched studies, but the studies differed in their prespecified endpoints and sample-size rationales. Downey et al. (2018a) enrolled 226 participants in a pilot cluster trial without a formal efficacy sample-size calculation, whereas Downey et al. (2020) based its sample size on feasibility parameters. The Weenk reports focused on feasibility, measurement comparison, or user experience (Weenk et al., 2017, 2019, 2020). Verrillo et al. (2019) reported a power calculation for its single-unit before–after quality-improvement comparison.

The larger randomized studies were designed around different primary endpoints. Mølgaard et al. (2025) enrolled 400 participants for the cumulative duration of severe vital-sign deviations. Khanna et al. (2026) planned approximately 500 participants per group for cumulative physiological deviation and analyzed 798 participants. Posthuma et al. (2024) evaluated new disability and stopped at 57% of planned enrollment because of COVID-19-related disruption.

Observational and before–after studies reported favorable associations in selected settings. Eddahchouri et al. (2022) reported lower unplanned ICU admission and rapid-response call rates after implementation, whereas the related ICU-transfer analysis found no difference in SOFA or APACHE severity at transfer (Eddahchouri et al., 2023). Vroman et al. (2023) reported fewer ICU transfers, shorter length of stay, and lower costs after implementation. Verrillo et al. (2019) reported a decrease in complications from 22% to 5.9%, with no failure-to-rescue events during the intervention period. In propensity-matched analyses, intermittent monitoring was associated with higher odds of the composite outcome than continuous monitoring in surgical patients (OR 3.42, 95% CI 3.19–3.67) and medical patients (OR 2.79, 95% CI 1.89–4.25) (Rowland et al., 2024, 2025).

Randomized findings varied by endpoint. Mølgaard et al. (2025) found no significant reduction in the prespecified composite duration of severe deviations, although time with SpO_2_ below 88% was lower and adverse events were numerically less frequent. Khanna et al. (2026) reported approximately 30 fewer minutes with SpO_2_ below 90% over 48 h, without significant differences in hypotension or tachycardia duration. Posthuma et al. (2024) reported new disability in 39.1% of the intervention group and 43.7% of controls. Study objectives, sample-size rationales, and endpoint-level interpretability are summarized in **Supplementary Table 6**.

### 3.7 Patient selection, monitoring exposure, and user experience

Acceptability was evaluated in populations with different eligibility criteria and monitoring durations. Several studies required adult participants who could provide consent and excluded cognitive impairment, delirium, palliative care, skin allergy, pacemakers, or short expected admissions. These criteria may underrepresent patients who are most difficult to monitor or respond to. Exposure ranged from approximately 24-72 hours in the Weenk studies to a median of 81 hours in Leenen et al. (2021), up to five postoperative days in Posthuma et al. (2024) and Mølgaard et al. (2025), and routine admission-based use in implementation studies. In Downey et al. (2018a), 34 of 142 intervention participants stopped monitoring early; patient request, discomfort, pruritus, and rash were reported. In Eddahchouri et al. (2022), the device was removed in 21% of monitored patients, including because of hyperactive delirium, skin problems, or refusal.

Patients generally described monitoring as acceptable and reassuring, particularly overnight, but emphasized that remote monitoring should not replace direct nurse-patient contact (Downey et al., 2018b; Weenk et al., 2020; Lockhorst et al., 2025). Clinician experience depended on alarm visibility and workflow. ViSi Mobile generated visible and audible alerts on patient and ward displays, whereas Sensium systems commonly delivered alerts remotely to nurse mobile devices. Alarm-free Healthdot implementations required scheduled trend review rather than local audible alarms. Nurses valued earlier visibility and potential time savings but reported false alerts, connectivity problems, battery replacement, device size, interpretation demands, and uncertainty about responsibility. Longitudinal implementation findings showed that acceptance could improve with experience, while adherence could decline when perceived added value was limited or implementation support diminished (Leenen et al., 2023; van Noort et al., 2024). Study-level selection, exposure, removal, and alarm context are reported in **Supplementary Table 7**.

### 3.8 Evidence map and translational pathway

**Figure 3:**
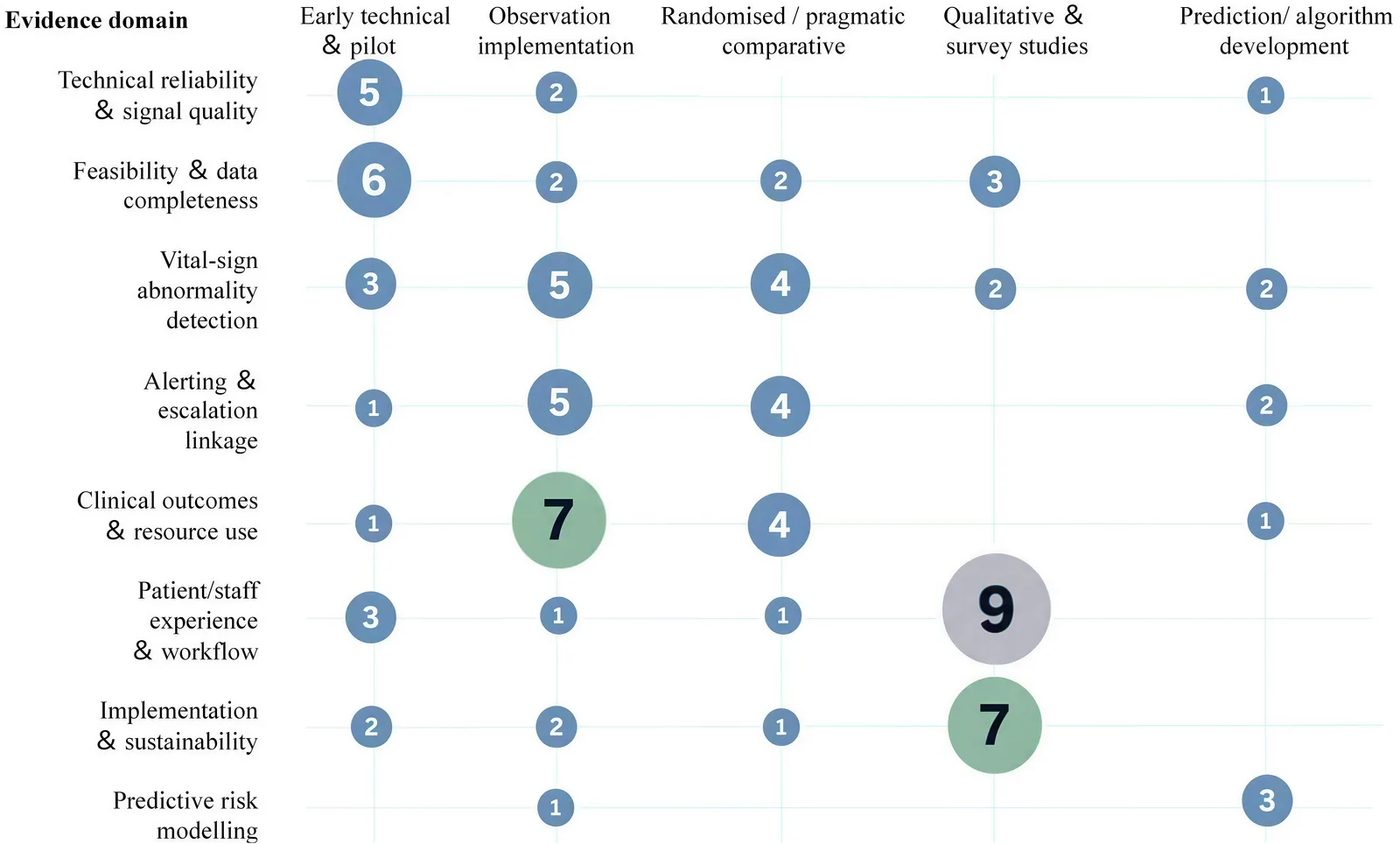
Matrix summarizing the number of included studies stratified by evidence domain and study design type. Studies may contribute to more than one evidence domain; therefore, bubble counts are not additive. This category includes offline algorithmic warning evaluations as well as one formal prediction-model study; it should not be interpreted as a mature prediction-model evidence base.

**Figure 3** summarizes the distribution of studies across evidence domains and study designs. Studies most frequently addressed clinical data acquisition, signal processing and data management, interpretation of physiological abnormalities, and user acceptability. Fewer studies reported alarm actionability, bedside reassessment, intervention, escalation, response fidelity, hard clinical endpoints, or economic outcomes.

Several validation and feasibility studies did not include a real-time clinical response pathway. Comparative studies frequently retained intermittent observations and, in some settings, introduced training, workflow modification, or escalation support alongside continuous monitoring. Across the six-stage monitor–response framework, more reports addressed the four afferent stages than the two efferent stages. A phase-level synthesis is provided in **Supplementary Table 8**.

## 4 Discussion

### 4.1 Evidence maturity across the afferent-efferent pathway

The central finding is not a lack of sensing capability, but an incomplete translational chain between abnormality detection and completed clinical response. Continuous monitoring identified hypoxemia and abnormal respiratory-rate episodes missed by scheduled observations, yet relatively few studies reconstructed whether a notification reached an accountable clinician, prompted bedside reassessment, led to intervention or escalation, and altered outcomes. **Figure 3** therefore indicates that the evidence base is more mature for the afferent limb than for the efferent limb.

This distinction helps explain why additional detection did not consistently translate into reductions in ICU transfer, disability, or mortality. Wearable monitoring is a complex service intervention: a technically accurate sensor may have limited clinical effect if alerts are not received, reassessed, or escalated. Conversely, outcomes may improve through training, heightened awareness, revised escalation practice, or additional staff support even when the sensor is not the sole active component. Future evaluations should define the pre-existing standard of care and report each step from alert generation to completed clinical action.

### 4.2 Measurement performance and comparator limitations

The apparent advantage of heart-rate over respiratory-rate performance should be read as a pattern within the included sensor-comparator pairs, not a universal hierarchy of wearable parameters. Heart rate was generally more consistent in the charted validation studies, whereas respiratory-rate results varied by device, sensing principle, movement, posture, and algorithm. The comparator also matters. Manual respiratory-rate measurement is affected by counting duration, rounding, observer variability, and changes in breathing when a patient is observed. Disagreement with routine care can therefore arise from the wearable device, the reference method, or both. Studies using an ICU-grade reference monitor provide a different level of technical inference from studies using routine nurse observations.

Technical feasibility should likewise be separated from parameter-level validity. A system can be deployable and acceptable while producing incomplete respiratory-rate data, connection failures, or repeated technical alerts. Reporting should distinguish data availability, artefact, clinically usable data, and measurement agreement for each parameter. Without this separation, ward feasibility may be misread as evidence of diagnostic accuracy.

### 4.3 Alarm design, actionability, and response fidelity

Alarm burden is not a fixed property of the device; it depends on thresholds, persistence requirements, annunciation delay, artefact filtering, adjustment rules, delivery route, and the method used to adjudicate true alerts. The findings of Leenen et al. (2021) illustrate why technical and physiological alerts must be reported separately and why predictive value should be accompanied by its numerator, denominator, and adjudication method.

The included studies also demonstrate that different designs trade immediacy against workload. Persistent-threshold alerts can suppress transient deviations, but may still generate technical burden. Algorithmic scores may provide longer lead time but low positive predictive value, as shown by the high number needed to evaluate in Peelen et al. (2023). Alarm-free trend review reduced immediate alert burden but shifted responsibility to scheduled nurse review and documentation. These approaches cannot be compared meaningfully unless studies report alert configuration, exposure time, alerts per patient-day, acknowledgement, bedside assessment, and resulting intervention. Response fidelity should be treated as an outcome in its own right.

### 4.4 Clinical outcomes, statistical capacity, and co-interventions

Clinical outcome evidence remains heterogeneous because the studies asked different questions. Pilot and feasibility studies were designed to establish recruitment, adherence, missing data, usability, or physiological detection, not to determine effects on death, cardiac arrest, unplanned ICU admission, or failure to rescue. A non-significant result for an uncommon endpoint in an underpowered study is absence of conclusive evidence, not evidence that the intervention has no effect. The converse is equally important: favorable numerical differences in a pilot study do not establish effectiveness when no adequately powered comparison was prespecified.

Observational and before–after studies often reported larger outcome differences than randomized studies. These differences may reflect benefits under mature implementation, but they are also compatible with secular trends, case mix, residual confounding, revised escalation practice, and concurrent training or technical support. Propensity matching reduces measured confounding but cannot remove all unmeasured differences. Randomized trials provide stronger protection against confounding, yet several were powered for physiological exposure or patient-reported disability rather than uncommon hard outcomes, and one was terminated early. The most defensible interpretation is that selected physiological and process benefits are supported, whereas effects on uncommon clinical outcomes remain uncertain.

### 4.5 Patient selection, workflow, and sustainability

Patient acceptability was generally favorable, but external validity is limited by consent-based recruitment, exclusions for cognitive impairment or skin intolerance, and variable monitoring duration. Short-term postoperative exposure in willing participants is not equivalent to sustained use throughout a complex medical admission. Device removal because of delirium, discomfort, rash, refusal, procedures, battery depletion, or technical failure should be reported alongside satisfaction scores, because these events identify populations and contexts in which implementation may be least reliable.

For clinicians, sustainability depended on whether the system reduced uncertainty without transferring unmanageable surveillance work to nurses. Training, key-user models, bedside coaching, technical support, electronic record integration, and feedback cycles improved early adoption in several reports. However, fidelity declined over time in a medical ward when perceived added value was limited. Preserving face-to-face assessment is also essential: patients valued remote surveillance as an additional safety layer, not as a substitute for nursing contact.

### 4.6 Strengths and limitations

This review used a scoping design suited to a heterogeneous and rapidly developing field, searched four major databases, and organized evidence across the complete monitor-response pathway rather than treating wearable monitoring as a stand-alone device. The extraction framework distinguishes technical from physiological alerts, documents comparator methods, charts standard care and response pathways, and relates clinical interpretations to each study’s prespecified endpoint and sample-size rationale.

Several limitations remain. No formal risk-of-bias assessment was undertaken, so the map should not be interpreted as a graded effectiveness review. The search did not include CINAHL, IEEE Xplore, trial registries, conference proceedings, or a systematic gray-literature search. Alarm thresholds, delays, individualized settings, standard care, response pathways, and fidelity were frequently not reported, limiting reconstruction of the complete intervention. Several reports selected participants willing and able to wear the technology and monitored them for only part of the admission. Many studies were not designed or powered for uncommon clinical outcomes. Predictive modeling was represented by one report, and the search was not designed as a prediction-model review; broader conclusions about predictive analytics are therefore not supported. Device versions, ward staffing, local escalation protocols, and health-system context also limit transferability.

### 4.7 Implications for practice and future research

Current evidence supports selective use cases in which the target abnormality, alert logic, accountable responder, and escalation action are explicit. Adoption should be preceded by local validation of connectivity, parameter completeness, alarm burden, and workflow capacity. Programs should retain a safety-net observation process until reliability is demonstrated, specify who owns each alert, and audit acknowledgement, bedside reassessment, escalation, and outcome. Economic evaluation should include implementation labor, training, devices, connectivity, alarm workload, avoided deterioration, and opportunity costs.

Future comparative studies should prespecify the primary clinical or physiological endpoint, justify sample size for that endpoint, report effect estimates with confidence intervals, and avoid interpreting underpowered null findings as proof of equivalence. Alarm studies should report physiological and technical alerts separately, thresholds, persistence, delays, adjustment rules, alerts per patient-day, positive predictive value, acknowledgement, and clinical action. Prediction modeling should be considered preliminary within this review: future models require external validation and prospective testing of calibration, actionability, workload, escalation behavior, and patient outcomes before deployment as decision support.

## 5 Conclusion

This scoping review maps an evidence base that is comparatively developed for clinical data acquisition, technical feasibility, data completeness, and detection of physiological abnormalities, but substantially less developed for clinical assessment, intervention, escalation, response fidelity, and patient or economic outcomes. Selected studies identified hypoxemia and abnormal respiratory-rate episodes not captured by intermittent observations. However, clinical outcome findings were heterogeneous, and several pilot, feasibility, validation, or implementation studies were not designed or powered to evaluate uncommon clinical endpoints. Incomplete reporting of standard care, alarm configuration, co-interventions, and response pathways further limited attribution of observed outcomes to the monitoring technology itself. Wearable monitoring should therefore be evaluated as a complex monitor-response intervention rather than as a stand-alone sensing technology.

## Data Availability

The raw data supporting the conclusions of this article will be made available by the authors, without undue reservation.
