# Supplementary material for: From perception to response: translational progress implementation challenges of wearable non-invasive continuous vital sign monitoring in early warning for inpatients
Source: Front Physiol. 2026 Sep 14;17:1925052. doi: 10.3389/fphys.2026.1925052 (PMC13589808; doi:10.3389/fphys.2026.1925052)
Supplement: Supplementary file 1 [file Supplementaryfile1.docx]

**Supplementary Materials**

From Perception to Response: Translational Progress and Implementation Challenges of Wearable Non-Invasive Continuous Vital Sign Monitoring in Early Warning for Inpatients

**Table S1. Database search strategies for the scoping review**

| **Database** | **Search Strategy** | **Records Retrieved** |
| --- | --- | --- |
| PubMed | ("Wearable Electronic Devices"[Mesh] OR "Monitoring, Physiologic"[Mesh] OR wearable*[tiab] OR wireless[tiab] OR biosensor*[tiab] OR sensor*[tiab] OR patch*[tiab] OR "wearable device*"[tiab] OR "wearable sensor*"[tiab] OR "wireless device*"[tiab] OR "wireless sensor*"[tiab] OR "remote monitor*"[tiab] OR "remote monitoring"[tiab] OR "continuous monitor*"[tiab] OR "continuous surveillance"[tiab])  AND  ("Vital Signs"[Mesh] OR "vital sign"[tiab] OR "vital signs"[tiab] OR "heart rate"[tiab] OR "respiratory rate"[tiab] OR "oxygen saturation"[tiab] OR SpO₂[tiab] OR temperature[tiab] OR "blood pressure"[tiab] OR "physiological monitoring"[tiab] OR "physiologic monitoring"[tiab])  AND  ("Inpatients"[Mesh] OR "Hospitalization"[Mesh] OR "Hospital Units"[Mesh] OR inpatient*[tiab] OR hospital*[tiab] OR ward*[tiab] OR "general ward*"[tiab] OR "medical ward*"[tiab] OR "surgical ward*"[tiab] OR postoperative[tiab] OR surgery[tiab] OR surgical[tiab])  AND  (deteriorat*[tiab] OR "clinical deterioration"[tiab] OR "early warning"[tiab] OR NEWS[tiab] OR NEWS2[tiab] OR MEWS[tiab] OR "rapid response"[tiab] OR "rapid response system"[tiab] OR "rapid response team"[tiab] OR "ICU transfer"[tiab] OR "unplanned ICU"[tiab] OR "intensive care unit admission"[tiab] OR "patient safety"[tiab] OR complication*[tiab] OR "adverse event*"[tiab] OR "clinical outcome*"[tiab] OR "patient outcome*"[tiab] OR "failure to rescue"[tiab] OR alarm*[tiab] OR alert*[tiab] OR notification*[tiab] OR implementation[tiab] OR workflow[tiab] OR feasibility[tiab] OR acceptability[tiab] OR usability[tiab] OR experience*[tiab] OR perception*[tiab] OR perspective*[tiab] OR nurs*[tiab])  AND  ("2010/01/01"[Date - Publication] : "2026/05/31"[Date - Publication]) | 3,723 |
| Web of Science | TS = ((wearable* OR wireless OR biosensor* OR sensor* OR patch* OR "wearable device*" OR "wearable sensor*" OR "wireless device*" OR "wireless sensor*" OR "remote monitor*" OR "remote monitoring" OR "continuous monitor*" OR "continuous surveillance")  AND  ("vital sign*" OR "heart rate" OR "respiratory rate" OR "oxygen saturation" OR SpO₂ OR temperature OR "blood pressure" OR "physiological monitoring"OR "physiologic monitoring")  AND  (inpatient* OR hospital* OR ward* OR "general ward*" OR "medical ward*" OR "surgical ward*" OR postoperative OR surgical OR surgery)  AND  (deteriorat* OR "clinical deterioration" OR "early warning" OR NEWS OR NEWS2 OR MEWS OR "rapid response" OR "rapid response system" OR "rapid response team" OR "ICU transfer" OR "unplanned ICU" OR "intensive care unit admission" OR "patient safety" OR complication* OR "adverse event*" OR "clinical outcome*" OR "patient outcome*" OR "failure to rescue" OR alarm* OR alert* OR notification* OR implementation OR workflow OR feasibility OR acceptability OR usability OR experience* OR perception* OR perspective* OR nurs*)) | 1,773 |
| Scopus | TITLE-ABS-KEY  ((wearable* OR biosensor* OR patch* OR "continuous monitoring" OR"wireless monitoring")  AND  ("vital sign*" OR "heart rate" OR "respiratory rate" OR SpO₂ OR "oxygen saturation" OR temperature OR "blood pressure")  AND  (hospitalized OR inpatient* OR "general ward*" OR postoperative)  AND  ("early warning" OR NEWS OR NEWS2 OR MEWS OR "deterioration")) | 171 |
| Embase | ('wearable device'/exp OR wearable*:ti,ab,kw OR 'wearable sensor':ti,ab,kw OR 'wearable sensors':ti,ab,kw OR 'wireless monitoring':ti,ab,kw OR 'wireless vital sign monitoring':ti,ab,kw OR 'wireless vital signs monitoring':ti,ab,kw OR 'continuous vital sign monitoring':ti,ab,kw OR 'continuous vital signs monitoring':ti,ab,kw OR 'continuous monitoring of vital signs':ti,ab,kw)  AND  ('vital sign'/exp OR 'heart rate'/exp OR 'respiratory rate'/exp OR 'oxygen saturation'/exp OR 'blood pressure'/exp OR 'body temperature'/exp OR 'vital sign':ti,ab,kw OR 'vital signs':ti,ab,kw OR 'heart rate':ti,ab,kw OR 'respiratory rate':ti,ab,kw OR 'oxygen saturation':ti,ab,kw OR SpO₂:ti,ab,kw OR temperature:ti,ab,kw OR 'blood pressure':ti,ab,kw)  AND  ('hospital patient'/exp OR inpatient*:ti,ab,kw OR hospital*:ti,ab,kw OR ward*:ti,ab,kw OR 'general ward':ti,ab,kw OR 'general wards':ti,ab,kw OR 'medical ward':ti,ab,kw OR 'medical wards':ti,ab,kw OR 'surgical ward':ti,ab,kw OR 'surgical wards':ti,ab,kw OR postoperative:ti,ab,kw)  AND  (deteriorat*:ti,ab,kw OR 'clinical deterioration':ti,ab,kw OR 'early warning':ti,ab,kw OR news:ti,ab,kw OR news2:ti,ab,kw OR mews:ti,ab,kw OR 'rapid response':ti,ab,kw OR 'rapid response team':ti,ab,kw OR 'icu transfer':ti,ab,kw OR 'unplanned icu':ti,ab,kw OR 'patient safety':ti,ab,kw) | 426 |

**Note:** This search covers literature records published from January 2010 to May 2026, with the final search completed in May 2026. Search terms were adapted according to the indexing rules and field tags of each database. An asterisk (*) indicates truncation, while quotation marks denote phrase or multi-word term searches. [tiab] refers to the title/abstract field in PubMed; TS denotes the Topic field in Web of Science; TITLE-ABS-KEY corresponds to the title, abstract, and keyword fields in Scopus; ti, ab, kw represents the title, abstract, and keyword fields in Embase. PubMed searches used Medical Subject Headings (MeSH), while Embase employed expanded subject headings (/exp) where applicable. Synonyms under the same concept were combined using OR logic, whereas the four core concepts-wearable monitoring or wireless monitoring, vital signs, hospital setting, and deterioration or early warning outcomes—were linked by AND logic. Before deduplication, the total number of retrieved records across all databases was 6,093. Abbreviations used in the search terms include ICU, intensive care unit; MEWS, Modified Early Warning Score; NEWS, National Early Warning Score; NEWS2, National Early Warning Score 2; and SpO₂, peripheral oxygen saturation.

**Table S2. Summary of included reports: design, setting, population, and monitoring devices**

| **Study (First Author, Year)** | **Study Design** | **Clinical Setting** | **Sample size / participants** | **Primary Device** | **Parameters** |
| --- | --- | --- | --- | --- | --- |
| Weenk 2017 | Prospective observational | Medical & surgical wards | 20 | ViSi Mobile, HealthPatch | HR, RR, SpO₂, BP, Temp |
| Weenk 2019 | Quantitative study within an RCT / method-comparison study | Medical & surgical wards | 60 | ViSi Mobile, HealthPatch | HR, RR, SpO₂, BP, Temp |
| Weenk 2020 | RCT | Medical & surgical wards | 90 | ViSi Mobile, HealthPatch | HR, RR, SpO₂, BP, Temp |
| Downey 2018a | Cluster RCT | Surgical wards | 226 | SensiumVitals | HR, RR, Temp |
| Duus 2018 | Prospective observational | Abdominal surgery | 50 | Isansys Lifetouch, Nonin | HR, RR, SpO₂ |
| Downey 2022 | Realist theory elicitation / implementation study | Surgical wards | Qualitative | SensiumVitals | HR, RR, Temp |
| Eddahchouri 2022 | Before-after | Medical & surgical wards | 2,466 / 2,303 | ViSi Mobile | HR, RR, SpO₂, BP |
| Haahr-Raunkjaer 2022 | Prospective cohort | Abdominal surgery | 500 | Isansys, Nonin, Meditech | HR, RR, SpO₂, BP |
| Leenen 2021 | Prospective cohort | Surgical wards | 30 | SensiumVitals | HR, RR, Temp |
| Leenen 2022a | Qualitative | Surgical wards | 12 nurses | SensiumVitals | HR, RR, Temp |
| Leenen 2022b | Mixed methods | Surgical wards | 56 | Philips Biosensor | HR, RR |
| Leenen 2023 | Mixed methods | Medical & surgical wards | 358 | Philips Healthdot | HR, RR |
| Leenen 2024 | Before-after | Surgical wards | 908 | Philips Healthdot | HR, RR |
| Mølgaard 2025 | RCT | Surgical wards | 400 | Isansys, Nonin, A&D | HR, RR, SpO₂, BP |
| Rowland 2024 | Propensity-matched | Surgical wards | 34,636 | ViSi Mobile | HR, RR, SpO₂, BP, ECG |
| Rowland 2025 | Propensity-matched | Medical wards | 7,971 | ViSi Mobile | HR, RR, SpO₂, BP, ECG |
| Khanna 2026 | Cluster RCT | Surgical wards | 798 | ViSi Mobile | HR, RR, SpO₂, BP, ECG |
| Becking-Verhaar 2023 | Cross-sectional survey | Medical & surgical wards | 58 nurses | ViSi Mobile | HR, RR, SpO₂, BP, Temp |
| van Noort 2024 | Qualitative | Surgical wards | 15 nurses/patients | ViSi Mobile | HR, RR, SpO₂, BP, Temp |
| Breteler 2020 | Method comparison | Surgical step-down | 25 | SensiumVitals, HealthPatch, EarlySense, Masimo | HR, RR |
| Vroman 2023 | Retrospective before-after | Surgical wards | 855 | Philips Biosensor | HR, RR |
| Posthuma 2024 | Stepped-wedge cluster RCT | Surgical wards | 747 | SensiumVitals | HR, RR, Temp |
| Peelen 2023 | Prospective cohort | Medical & surgical wards | 1,529 | ViSi Mobile | HR, RR, SpO₂, BP |
| van Rossum 2021 | Qualitative | Esophageal surgery | 12 staff | Conceptual interview | HR, RR, Temp, SpO₂ |
| Lockhorst 2025 | Prospective survey | Surgical wards | 298 | SensiumVitals | HR, RR, Temp |
| Scheid 2025 | Deep learning modeling | Medical & surgical wards | 888 | VitalConnect, Biobeat | HR, RR, SpO₂, Temp |
| Eddahchouri 2023 | Before-after | Medical & surgical wards | 152 ICU transfers | ViSi Mobile | HR, RR, SpO₂, BP |
| Downey 2018b | Qualitative interviews | Surgical wards | 12 patients | SensiumVitals | HR, RR, Temp |
| Downey 2019 | Clinical validation | Surgical wards | 51 | SensiumVitals | HR, RR, Temp |
| Kooij 2022 | Qualitative | Teaching hospitals | 16 nurses | Philips / Sensium | HR, RR |
| Verrillo 2019 | QI before-after | Orthopedic/trauma | 427 / 422 | ViSi Mobile | HR, RR, SpO₂, BP, Temp |
| Downey 2020 | Feasibility RCT | Surgical wards | 136 | SensiumVitals | HR, RR, Temp |

**Note:** BP, blood pressure; ECG, electrocardiogram; HR, heart rate; QI, quality improvement; RCT, randomized controlled trial; RR, respiratory rate; SpO₂, peripheral oxygen saturation; Temp, temperature. Multiple reports from the same research program were retained when they reported distinct outcomes, populations, or implementation perspectives.

**Table S3. Technical validation, reference/comparator methods, and data completeness**

| **Study** | **Device/parameter** | **Reference or comparator** | **Analysis/configuration** | **Main technical finding** | **Completeness and interpretive limitation** |
| --- | --- | --- | --- | --- | --- |
| Weenk 2017 | ViSi Mobile and HealthPatch; HR, RR, SpO₂, BP, Temp | Routine nurse observations; pulse/SpO₂/BP/temperature by ward methods and RR by routine manual observation | Paired ward observations; study-specific acceptable discrepancy ranges | Mean differences were generally small, but clinically relevant MEWS differences occurred in 15% for ViSi Mobile and 27% for HealthPatch, mainly because of RR disagreement | Mean exposure 61.9 h (ViSi Mobile) and 57.5 h (HealthPatch). Connectivity failure caused 70% of ViSi Mobile artefacts. Nurse RR was not an error-free reference. |
| Weenk 2019 | ViSi Mobile and HealthPatch; multiparameter MEWS | Regular nurse MEWS observations during 24–72 h continuous monitoring | Device data compared with nurse observations; individualized normal ranges displayed but clinical staff were blinded to device results | Median device-derived MEWS was higher than nurse MEWS, predominantly because of RR differences; 71 and 32 high-MEWS periods were identified between nurse observations | Missing data 10.1% for ViSi Mobile and 8.4% for HealthPatch. Comparison was retrospective and based on routine ward measurements. |
| Downey 2019 | SensiumVitals; HR, RR, Temp | Routine NEWS observations: pulse from pulse oximeter, tympanic temperature, and manually counted RR | Paired median sensor values within +/-10 min; bias and 95% limits of agreement | Small mean HR bias but wide limits; RR and temperature agreement were outside study-specific acceptable limits | Usable data: HR 59.2%, RR 34.1%, Temp 72.8%. Manual RR distributions were sometimes clinically implausible, limiting attribution of disagreement solely to the sensor. |
| Breteler 2020 | SensiumVitals, HealthPatch, EarlySense, Masimo Radius-7; HR and RR | ICU-grade XPREZZON monitor in a surgical step-down unit | Method-comparison bias, 95% limits of agreement, and clinical error-grid analysis | HR was generally acceptable across systems; RR performance varied and HealthPatch overestimated RR by about 4.4 breaths/min | Device-specific data loss approximately 6%-38%; EarlySense produced no data during bed exit. Stronger reference standard than routine manual observations. |
| Duus 2018 | Isansys Lifetouch and Nonin WristOx; HR, RR, SpO₂ | Routine EWS observations; comparison addressed detection rather than sensor accuracy | Continuous monitoring across the first postoperative days | Continuous monitoring detected SpO₂ <92% in 98% versus 16% by routine EWS; SpO₂ <85% for >10 min occurred in 52% | HR/RR availability about 89.2%; SpO₂ availability about 65.3%. The comparison shows observation-density differences, not validation against a gold standard. |
| Leenen 2021 | SensiumVitals; HR, RR, Temp | No independent continuous reference; nurse MEWS and electronic record used for alert adjudication | Thresholds HR 50–120/min, RR 8–24/min, axillary Temp 34.5–38.5 °C; persistence ≥14 min | Ward deployment was feasible, but parameter-specific artefact was substantial | Median monitoring 81 h. Artefact: HR 19%, RR 51%, Temp 9%. Feasibility did not imply complete or uniformly valid data. |
| Leenen 2022b/2023 | Philips Biosensor/Healthdot; HR and RR trends | Standard intermittent manual observations continued; no automated alarm reference comparison | Hourly dynamic EWS and scheduled nurse trend review twice per shift | Trend monitoring was operationally feasible without automated alarms; 17 abnormal trends were identified in the pilot | Ten percent of sensors were replaced prematurely in the 358-patient implementation. These studies evaluated workflow/fidelity, not measurement accuracy. |
| Mølgaard 2025 | WARD-CSS multimodal sensors; severe vital-sign deviations | Standard intermittent NEWS plus blinded continuous data in the control group | Prespecified physiological thresholds and persistence filtering; cumulative duration of severe deviations | No significant reduction in the primary composite duration; SpO₂ <88% was reduced by 47 min/day | Monitoring median 2 d 20 h intervention and 2 d 3 h control; maximum 5 days. This was an outcome trial, not a device‑accuracy study. |

**Note:** Comparator quality varied substantially. Routine manual respiratory-rate observations are subject to rounding and observer variability and should not be interpreted as an error-free reference. BP, blood pressure; EWS, early warning score; HR, heart rate; MEWS, Modified Early Warning Score; NEWS, National Early Warning Score; RR, respiratory rate; SpO₂, peripheral oxygen saturation; Temp, temperature.

**Table S4. Alarm-system design, alarm burden, and clinical actionability**

| **Study** | **Alert type and thresholds** | **Persistence/delay and adjustment** | **Delivery/location** | **Burden or predictive value** | **Clinical assessment/action** |
| --- | --- | --- | --- | --- | --- |
| Weenk 2017 | Out-of-range physiological alarms; exact cutoffs not fully reported | Device-specific logic; adjustment details NR | Toughbook/cloud display; study observer informed nurse | Technical artefacts included 70% connectivity failure for ViSi Mobile; alerts/day NR | Nurse review; one reported deterioration led to further evaluation |
| Weenk 2019 | Red physiological and blue technical alarms; normal ranges individualized | Delay/persistence NR | Toughbook at nurse post, but clinicians were blinded for study purposes | 71 and 32 high-MEWS periods between nurse observations; not an alarm-burden trial | No real-time action evaluated |
| Downey 2018a/2020 | Preset physiological norms for HR, RR, Temp | Initial burden judged unacceptable; thresholds and reminder intervals adjusted, reducing alerts by about 90% in pilot implementation | Central station/ward computer and nurse mobile device | Alerts per patient-day and PPV NR | Nurse assessment; treatment decisions according to local protocols |
| Leenen 2021 | Physiological: HR 50–120/min, RR 8–24/min, Temp 34.5–38.5 °C; technical/system alerts classified separately | Deviation ≥14 min; reminders until acknowledgement | Nurse mobile application and desktop | 972 total: 878 system (90.3%), 94 physiological (9.7%); median 4.5/patient-day; true positive 33/94 (35.1%), false 41, indeterminate 20 | Nurse manual MEWS and clinical review; true/false adjudicated retrospectively by two reviewers |
| Leenen 2022b/2023 | No automated physiological alarms; dynamic trend score | Scheduled review every 4 h/twice per shift; individualized thresholds not applicable | Digital platform and EHR documentation | No alarm rate; 17 abnormal trends in pilot; fidelity 80.5% in pilot and 70.7% in implementation | Nurse assessment/documentation; physician review during rounds; actions recorded for deviations |
| Eddahchouri 2022 | Single-channel physiological alarms outside preset safety limits; default limits adjustable by clinicians | Delay/persistence not adequately reported | Visible/audible patient and remote ward displays | Alerts per patient-day and PPV NR | Nurse checked trend and assessed patient; physician/RRT according to MEWS protocol |
| Posthuma 2024 | HR <50 or >120/min; RR <8 or >24/min; Temp >39 °C; system disconnection displayed | Deviation >14 min; nurses could optimize limits after patient assessment | Audio notification to nurse handheld device | Alarm burden/PPV NR | Assess alarm and patient; if no innocuous cause, measure full MEWS and follow escalation protocol |
| Mølgaard 2025 | NEWS-based severe deviation thresholds for SpO₂, RR, HR, and BP | Algorithm used persistence/duration filters; patient-specific adjustment NR | Staff smartphones | Detailed alerts/day and PPV not the primary outcome; 28% lacked documented acknowledgement/response | Existing escalation actions; no intervention mandated by the device |
| Khanna 2026 | MAP <65 mmHg, SpO₂ <90%, HR >110/min | Persistence/delay details not fully reported in main report; no patient-specific adjustment reported | Central station and assigned nurse phones; escalation to other nurses/unit manager | HR >110 and SpO₂ <90 were the most frequent alerts; standardized rate/PPV NR | Protocol-based nursing escalation; resulting bedside interventions not reliably captured |
| Peelen 2023 | VSI ≥3 versus periodic EWS ≥6 | Warning period ended after 4 h and extended by subsequent alerts; offline scenario | No live delivery in study analysis | VSI 0.99 vs EWS 0.13 warnings/patient-day; PPV 0.007 vs 0.048; NNE 152 vs 21 | No real-time clinical assessment/action evaluated |
| Verrillo 2019 | Parameter alerts included SBP, high RR, and low SpO₂; full threshold set NR | Adjustment/delay NR | Ward continuous monitoring system | Reported PPV: SBP 97%, high RR 85%, low SpO₂ 76%; denominators not fully presented in manuscript table | Nurses integrated alerts with assessment; action-level denominator NR |

**Note:** BP, blood pressure; EWS, early warning score; HR, heart rate; MAP, mean arterial pressure; MEWS, Modified Early Warning Score; NNE, number needed to evaluate; NR, not reported; PPV, positive predictive value; RR, respiratory rate; SBP, systolic blood pressure; SpO₂, peripheral oxygen saturation; Temp, temperature; VSI, Visensia Safety Index. The 35.1% true-positive rate in Leenen 2021 is 33/94 physiological alerts and does not include the 878 technical/system alerts.

**Table S5. Clinical monitoring context, response pathway, and implementation co‑interventions**

| **Study** | **Standard care and concurrent observations** | **Data recipient/reviewer** | **Alert or trend response and escalation** | **Training, protocol change, or co‑intervention** | **Adherence/response fidelity** |
| --- | --- | --- | --- | --- | --- |
| Weenk 2017 | Routine MEWS at least 3 times/day continued | Study staff viewed device data; nurse was informed when an alarm occurred | Nurse performed clinical assessment; one deterioration prompted further diagnostic and surgical care | Pilot deployment; device support and study observation | NR; response pathway not a formal study endpoint |
| Weenk 2019 | Regular nurse MEWS continued during 24–72 h monitoring | Device results used retrospectively; clinicians were blinded | No real-time clinical response intervention; high-MEWS periods were analyzed after monitoring | Individualized normal ranges could be displayed | Not applicable to clinical response |
| Downey 2018a | Usual intermittent NEWS continued in both groups | Central station or mobile device carried by the patient nurse | Nurse assessed the patient; junior doctor made treatment decisions within local protocols | Face-to-face nurse training; alert thresholds/reminder intervals changed after excessive early burden | Monitoring discontinued early in 34/142 intervention participants; response fidelity NR |
| Downey 2020 | Standard NEWS continued; intervention added SensiumVitals | Patient nurse mobile device and ward computer | Clinical response was not mandated and remained at nurse discretion within hospital protocols | Thorough nurse training before study | Feasibility outcomes included protocol adherence; efficacy response fidelity NR |
| Eddahchouri 2022 | Same MEWS protocol before/after: routine every 8 h, more frequently at higher scores | Patient wrist display and remote ward monitors; nurses verified values in EHR | Nurse/ward physician assessment; RRT consultation according to existing MEWS thresholds; no automatic RRT activation | Mandatory product training, superusers, on-ward support; no major staffing/policy change reported | Additional measurements increased; device removed in 21% for delirium, skin problems, refusal, or other reasons |
| Eddahchouri 2023 | Same MEWS and RRT protocol as before-after parent study | Ward nurses and existing escalation team | Escalation to ward physician/RRT at protocol thresholds | Same implementation context as parent study | Response fidelity NR; analysis included all unplanned ICU transfers |
| Leenen 2021 | Routine ward MEWS remained available | Mobile nurse application and desktop; reminders until acknowledgement | Nurse instructed to perform manual MEWS and act through standard escalation | 35 nurses trained; 10 key users received additional training and bedside support | Alert acknowledgement technically required; clinical response adjudicated retrospectively |
| Leenen 2022b | Standard intermittent MEWS once daily continued | Nurses reviewed digital trends; physicians reviewed during morning rounds | No automated alarms; trend review every 4 h/twice per shift, documentation, and escalation when abnormal | Implementation preparation and nurse instruction | Trend assessment documented in 80.5% of shifts; all 17 abnormal trends identified |
| Leenen 2023 | Standard intermittent manual observations continued | Nurses reviewed and reported trends each shift | No automated alarms; nurse activities recorded for deviating trends | Daily coaching early in implementation, key users, feedback dashboards, and improvement cycles | Overall fidelity 70.7%; surgery 73.6%, medicine 64.1%; medical-ward fidelity declined over time |
| Leenen 2024 | Intermittent standard care continued; continuous trends added | Ward nurses and house officers | Additional nursing activity or clinician contact based on trend review | Same mature Healthdot implementation program | 35% triggered additional nursing activity; nurse-to-house-officer calls fell from 15% to 8%; detailed response completion NR |
| Mølgaard 2025 | Standard intermittent NEWS in both groups | Real-time alerts to staff smartphones | No mandated intervention; nurses advised to use existing escalation actions and hospital protocols | Staff instruction, pretraining, and run-in period | In 28% of intervention cases no nurse acknowledgement/response was documented |
| Posthuma 2024 | Standard intermittent MEWS every 8 h or more frequently continued | Audible notification to nurse handheld device | Nurse assessed alarm/patient; innocuous causes required no action; otherwise full MEWS and hospital escalation protocol | System installation and ward training; alarm limits could be optimized after assessment | NR; trial stopped early because of COVID-19-related disruption |
| Khanna 2026 | All patients received intermittent vital-sign observations; continuous data visible only during unblinded periods | Central station and assigned nurse phone | If unaddressed, alert escalated to other floor nurses and then unit manager according to hospital protocol | Clinicians briefed on roles and protocol | Planned bedside intervention analysis was not reliable because nursing documentation was inadequate |
| Verrillo 2019 | Episodic vital-sign collection remained at standard unit times and as clinically needed | Ward nurses received continuous data | Nurses integrated trends with assessment and escalated according to unit practice | Education, daily coaching, and 24/7 clinical support during the first 6 weeks | Compliance increased from 38% to 62%; specific alert-to-action fidelity NR |
| Peelen 2023 | Periodic EWS remained in use although continuous monitoring was routine | No deployed recipient for study comparison; retrospective/offline modeling | No real-time response tested; warning periods compared with subsequent escalation-of-care events | No new clinical response protocol for the modeled comparison | Not applicable |
| Rowland 2024/2025 | Continuous and intermittent groups identified from routine clinical data | NR | NR; clinical response pathway and co‑interventions not reconstructed from administrative data | NR | NR |
| Vroman 2023 | Historical standard care versus later continuous-monitoring implementation | NR in sufficient detail for reconstruction | NR in sufficient detail | Before-after service implementation; concurrent changes incompletely separable | NR |

**Note:** EHR, electronic health record; MEWS, Modified Early Warning Score; NEWS, National Early Warning Score; NR, not reported; RRT, rapid response team. “NR” indicates that the primary report did not provide sufficient detail to reconstruct the item; missing reporting is itself an evidence gap.

**Table S6. Study purpose, sample-size rationale, and endpoint-level interpretability**

| **Study/design** | **Prespecified purpose or primary endpoint** | **Sample-size rationale/achieved sample** | **Powered for clinical endpoint discussed?** | **Key result and comparator direction** | **Interpretive boundary** |
| --- | --- | --- | --- | --- | --- |
| Weenk 2017 pilot feasibility | Feasibility, technical comparison, and user experience | No formal power; n=20 estimated sufficient because preliminary data were unavailable | No - not for hard clinical outcomes | Measurement and usability findings; one deterioration case | Clinical event is illustrative, not an effectiveness estimate |
| Weenk 2019 method comparison | MEWS agreement and high-MEWS periods between observations | No formal power; n=60 estimated sufficient | No | More high-MEWS periods detected by device data | Additional detection does not establish outcome benefit |
| Weenk 2020 RCT/user experience | Experiences, psychological distress, and usability | Sample informed by qualitative data saturation; 90 randomized | No | No difference in psychological distress; favorable usability | Not an effectiveness trial for ICU admission, mortality, or complications |
| Downey 2018a pilot cluster RCT | Time to antibiotics in sepsis; pilot practicality/acceptability | No formal efficacy calculation; target estimates 325–625; achieved 226 | No definitive efficacy power | Antibiotics 626 vs 1012.8 min; LOS 13.3 vs 14.6 d; overlapping uncertainty | Favorable numerical differences are exploratory |
| Downey 2020 feasibility RCT | Recruitment, randomization, adherence, missing data, and candidate endpoints | Target at least 120 based on feasibility parameters; achieved 136 | No | Feasibility achieved; clinical outcomes descriptive | Not designed to demonstrate clinical effectiveness |
| Eddahchouri 2022 before-after | Co-primary unplanned ICU admission and RRT calls | All eligible admissions in two periods; prospective power rationale NR | Unclear | ICU 3.4% to 2.3%; RRT 4.3% to 3.1% | Before-after association; residual confounding and implementation co‑interventions possible |
| Eddahchouri 2023 before-after | Illness severity at unexpected ICU transfer | All transfers: 93 before and 59 after; power rationale NR | Unclear/likely limited for modest differences | No significant SOFA/APACHE or mortality differences | Non-significance does not establish equivalence |
| Verrillo 2019 QI before-after | Complications, ICU transfer, RRT, and failure to rescue | Power analysis required 306; achieved 427 before and 422 after | Powered for project comparison, but not randomized | Complications 22% to 5.9%; RRT unchanged; no FTR in intervention | Single-unit temporal comparison; causal attribution limited |
| Vroman 2023 retrospective before-after | ICU transfer, LOS, and cost | n=855; prospective power rationale NR | Unclear | ICU transfer 13.2% to 5.9%; cost EUR 4,855 to 4,064 | Retrospective temporal comparison |
| Rowland 2024 propensity matched | Composite in-hospital mortality or ICU transfer | Large administrative cohort; prospective power calculation NR; n=34,636 | Large sample, but observational | Intermittent vs continuous OR 3.42 (95% CI 3.19–3.67) | Direction corrected; residual confounding remains |
| Rowland 2025 propensity matched | Composite mortality or ICU transfer in medical patients | n=7,971; matched continuous 1,329 vs intermittent 1,854; prospective power NR | Large sample, but observational | Intermittent vs continuous OR 2.79 (95% CI 1.89–4.25) | Direction corrected; association driven mainly by ICU admission |
| Mølgaard 2025 RCT | Cumulative duration of severe vital-sign deviations | Calculated for 50% reduction, 80% power, alpha .05; required 384, enrolled 400 | Yes for physiological primary endpoint; no for rare hard outcomes | Primary composite not significant; SpO₂ <88% reduced by 47 min/day | Hard outcomes were secondary and less precise |
| Posthuma 2024 stepped-wedge cluster RCT | New disability at 3 months | Planned sample not achieved; stopped at 57% enrollment because of COVID-19; n=747 | Underpowered relative to original plan | New disability 39.1% intervention vs 43.7% control | Early termination limits precision |
| Khanna 2026 cluster crossover RCT | Cumulative hypotension, hypoxemia, and tachycardia over initial 48 h | Planned about 500/group using ICC assumptions; analyzed 404 vs 394 | Below planned total; endpoint-specific power reduced | SpO₂ <90% reduced about 30 min/48 h; hypotension and tachycardia not significant | Null secondary components should not be read as proof of no effect |
| Peelen 2023 modeling cohort | Sensitivity, warning burden, NNE, and lead time for VSI vs EWS | n=1,529; sample-size rationale NR | Not a clinical-effectiveness trial | VSI sensitivity 55% vs EWS 51%; NNE 152 vs 21 | Offline warning comparison did not test response or outcomes |
| Scheid 2025 prediction model | Develop and validate deterioration prediction from continuous data | n=888; hard events were sparse; clinical deployment power not established | No for clinical effectiveness | AUC about 0.89 and lead time up to 17 h | One preliminary modeling report; not a deployed decision-support trial |

**Note:** APACHE, Acute Physiology and Chronic Health Evaluation; AUC, area under the curve; EWS, early warning score; FTR, failure to rescue; ICC, intracluster correlation coefficient; ICU, intensive care unit; LOS, length of stay; NNE, number needed to evaluate; NR, not reported; OR, odds ratio; RRT, rapid response team; SOFA, Sequential Organ Failure Assessment; SpO₂, peripheral oxygen saturation; VSI, Visensia Safety Index. No retrospective power calculations were performed. “Powered” refers to the original report’s sample-size rationale for the endpoint under discussion.

**Table S7. Patient selection, monitoring exposure, device removal, and local implementation context**

| **Study** | **Selection context** | **Monitoring exposure** | **Removal/incomplete exposure** | **Alarm visibility and local context** | **Implication for acceptability/generalizability** |
| --- | --- | --- | --- | --- | --- |
| Weenk 2017 | Adults on medical/surgical wards; expected stay ≥48 h; local-language participation | 2–3 days; mean 61.9 h ViSi Mobile and 57.5 h HealthPatch | Technical Wi-Fi/Bluetooth failures; no systematic removal denominator reported | Toughbook/cloud; nurses could be informed by observer | Small willing sample; short exposure limits generalization |
| Weenk 2019 | Adults; expected admission ≥3 days; short admissions excluded | 24–72 h; total 1,282 h ViSi Mobile and 1,886 h HealthPatch | Missing data 10.1% and 8.4% | Alarms displayed, but clinicians blinded | Usability/measurement evidence does not reflect routine response |
| Weenk 2020 | Randomized ward participants able to consent/interview | At least 24 h in intervention arms | Device-specific discomfort and practical barriers reported qualitatively | Device and remote displays; workflow varied | Consent and interview requirements may exclude less able patients |
| Downey 2018a | Adult surgical patients; consent-based pilot | Mean about 5 days, range 1–24; 75.7% wore ≥5 days | 34/142 stopped early: 23 patient request; rash, itching/discomfort, and other reasons | Remote nurse mobile and central station | Acceptability high among completers, but early discontinuation is clinically relevant |
| Downey 2020 | Elective major surgery; consent required | Entire stay or at least 5 days was an adherence target | Withdrawal before discharge, loss of capacity, clinical need, discomfort, and false alerts reported | Nurse mobile and ward computer | Feasibility population may differ from emergency/cognitively impaired patients |
| Eddahchouri 2022 | All adult admissions to selected medical/surgical wards unless monitoring unsuitable | Admission-based routine deployment | Device removed in 386 patients (21%), including delirium, skin problems, refusal, palliative care, or other reasons | Visible/audible patient wrist display and ward remote monitors | Real-world uptake identifies important exclusions and incomplete exposure |
| Leenen 2021 | Elective colorectal/pancreatic surgery; excluded cognitive impairment, emergency/palliative care, pacemaker, allergy, conflicting study | Median 81 h (IQR 47–143), generally until discharge | Sensor/data artefact; patient discontinuation uncommon; 93% acceptance | Remote mobile and desktop alerts | Selected elective sample may overestimate acceptability in complex medical patients |
| Leenen 2022b | Surgical ward patients suitable for biosensor monitoring | During ward stay; exact proportion of admission varied | Sensor/workflow interruptions reported | No audible automated alarm; remote trend platform | Acceptability reflects scheduled review model rather than alarm exposure |
| Leenen 2023 | 358 medical/surgical ward patients in routine implementation | 45,113 monitored hours over 6-month ward periods | 10.3% sensors replaced prematurely; temporary removal for procedures | No automated alarms; trend review each shift | Longitudinal fidelity provides stronger sustainability evidence, but ward differences were substantial |
| Mølgaard 2025 | Age ≥50, major noncardiac surgery, expected ≥2 nights; excluded inability/unwillingness, pacemaker/ICD, sensor allergy | Median 2 d 20 h intervention, 2 d 3 h control; maximum 5 days | Some alerts lacked documented response; device exposure did not cover every admission completely | Remote smartphone alerts; patients/outcome assessors blinded | High-risk elective/postoperative selection limits wider medical-ward generalization |
| Posthuma 2024 | Intermediate/high-risk surgical adults providing consent | Up to 5 days or discharge after PACU | Trial enrollment disrupted and terminated early during COVID-19 | Audible handheld nurse notification | Consent-based surgical sample and incomplete trial recruitment limit precision |
| Khanna 2026 | Postoperative adults; per-protocol high-risk subgroup included age/risk criteria | Initial 48 h/through third postoperative morning; all participants had some continuous data | Documentation prevented reliable assessment of interventions | Central station and nurse phone; escalation through unit hierarchy | Cluster crossover improves pragmatism, but response exposure was not completely documented |
| Lockhorst 2025 | Survey of 191 patients, 88 nurses, and 19 physicians after surgical-ward use | Exposure varied by admission; exact per-patient duration NR | NR | Local workflow and remote monitoring experience | Positive survey results may be influenced by respondent participation and variable exposure |
| van Noort 2024 | Key-user nurses and patients in one surgical unit three years after implementation | Long-term organizational exposure; individual patient duration NR | Device size, battery life, and Wi-Fi remained concerns | Existing ward alarm system; practices evolved with experience | Strong contextual depth but single mature ward limits transferability |

**Note:** ICD, implantable cardioverter-defibrillator; IQR, interquartile range; NR, not reported; PACU, post-anesthesia care unit. Reported acceptability should be interpreted alongside selection criteria, exposure duration, and incomplete monitoring.

**Table S8. Evidence maturity across the monitor-response pathway**

| **Monitor-response phase** | **What the evidence supports** | **Persistent limitation/efferent gap** | **Priority for future studies** |
| --- | --- | --- | --- |
| Clinical data acquisition (afferent) | Wearable systems can acquire HR, RR, SpO₂, temperature, and BP on general wards; HR was generally more consistent than RR within included validation studies | Accuracy and completeness are parameter-, sensor-, activity-, and comparator-dependent; routine manual RR is not an error-free reference | Use appropriate reference standards; report parameter-level bias, limits of agreement, artefact, and clinically usable data |
| Signal processing and data management (afferent) | Wireless transmission and longitudinal trend display were feasible in multiple wards | Connectivity failure, battery replacement, procedure-related removal, and incomplete RR/SpO₂ data remain common | Report monitoring exposure, missingness mechanisms, downtime, latency, and recovery procedures per patient-day |
| Modeling and interpretation (afferent) | Continuous data identify additional hypoxemia and abnormal RR episodes; one preliminary prediction model showed high discrimination | Detected episodes may be transient or non-actionable; model evidence is sparse and search was not prediction-focused | Validate externally; report calibration and subgroup performance; link predictions to prespecified actions |
| Alerting and notification (afferent) | Persistent-threshold, mobile, central, individualized, and alarm-free trend strategies have all been implemented | Technical alerts can dominate; thresholds, delays, adjustment, PPV, and denominators are often unreported | Use standardized alarm reporting: physiological vs technical, thresholds, persistence, delays, alerts/patient-day, PPV, acknowledgement |
| Clinical response and action (efferent) | Selected studies specified nurse assessment, manual MEWS/NEWS reassessment, physician review, or protocol escalation | Who owns the alert, response time, bedside assessment, intervention, and escalation completion are often unclear | Prespecify and audit the entire alert-to-action pathway; report response fidelity and reasons for non-response |
| Implementation and sustainability (efferent) | Training, key users, coaching, and workflow integration can support uptake; patients often report reassurance | Fidelity can decline; selection, device removal, workload, alarm burden, and loss of direct contact affect sustainability | Use longitudinal, multicenter implementation studies with workload, equity, acceptability, and unintended-consequence outcomes |
| Clinical and economic outcomes (efferent) | Some observational studies report fewer ICU transfers, complications, shorter stay, or lower cost; selected RCTs reduce hypoxemic burden | Randomized evidence is heterogeneous; several studies were not powered for rare outcomes; co‑interventions impede attribution | Power trials for prespecified outcomes; distinguish absence of evidence from evidence of absence; conduct full economic evaluations |

**Note:** BP, blood pressure; HR, heart rate; ICU, intensive care unit; MEWS, Modified Early Warning Score; NEWS, National Early Warning Score; PPV, positive predictive value; RR, respiratory rate; SpO₂, peripheral oxygen saturation. The evidence base is more mature for the afferent limb than for the efferent limb.
